# General control non-repressible 20 (GCN20) functions in root growth by modulating DNA damage repair in Arabidopsis

**DOI:** 10.1186/s12870-018-1444-9

**Published:** 2018-11-12

**Authors:** Tong-Tong Han, Wen-Cheng Liu, Ying-Tang Lu

**Affiliations:** State Key Laboratory of Hybrid Rice, College of Life Sciences, Wuhan University, Wuhan, 430072 China

**Keywords:** DNA damage repair, GCN20, Root elongation, Cell cycle, Root meristem

## Abstract

**Background:**

Most ABC transporters are engaged in transport of various compounds, but its subfamily F lacks transmembrane domain essential for chemical transportation. Thus the function of subfamily F remains further elusive.

**Results:**

Here, we identified General Control Non-Repressible 20 (GCN20), a member of subfamily F, as new factor for DNA damage repair in root growth. While *gcn20–1* mutant had a short primary root with reduced meristem size and cell number, similar primary root lengths were assayed in both wild-type and *GCN20::GCN20 gcn20–1* plants, indicating the involvement of GCN20 in root elongation. Further experiments with EdU incorporation and comet assay demonstrated that *gcn20–1* displays increased cell cycle arrest at G2/M checkpoint and accumulates more damaged DNA. This is possible due to impaired ability of DNA repair in *gcn20–1* since *gcn20–1* seedlings are hypersensitive to DNA damage inducers MMC and MMS compared with the wild type plants. This note was further supported by the observation that *gcn20–1* is more sensitive than the wild type when subjected to UV treatment in term of changes of both fresh weight and survival rate.

**Conclusions:**

Our study indicates that GCN20 functions in primary root growth by modulating DNA damage repair in Arabidopsis. Our study will be useful to understand the functions of non-transporter ABC proteins in plant growth.

**Electronic supplementary material:**

The online version of this article (10.1186/s12870-018-1444-9) contains supplementary material, which is available to authorized users.

## Background

Unlike animals, plants cannot change their location and thus plant roots in soil are constantly exposed to adverse environmental stresses such as high salinity, drought, free radicals, alkylating agents and heavy metals [1–5]. These adverse conditions damage DNA of the root meristem cells to affect genomic integrity and stability [6]. Root meristem accommodates stem cells that continually divide asymmetrically to produce new stem cells and daughter cells for root growth [7]. Therefore, the inhibition of the activity of root meristem by cell cycle restrains root growth and thus impairs plant growth and development [8–10].

DNA damages include base alkylation and oxidation, formation of abasic sites and pyrimidine dimers, DNA inter-strand crosslinks, single strand breaks (SSBs) and double strand breaks (DSBs) [11]. To cope with these DNA damages, eukaryotic cells trigger the DNA damage response (DDR) to maintain genome stability [5]. When cells undergo DNA damage, the activated cell cycle checkpoints transiently arrest cell cycle for lesion repair before the cell cycle continues. If the DNA damage is unrepairable, the cells will terminate cell division or suffer programmed cell death (PCD) [6]. Two kinases, ATM (Ataxia Telangiectasia mutated) and ATR (ATM and Rad3-related) function as main DDR regulators to coordinate cell cycle progression and activation of DNA repair pathways [4]. While ATM is mainly triggered by DSBs, ATR is activated by a broad range of lesions including UV photoproducts, DNA breaks and DNA crosslinks [12, 13]. In Arabidopsis, both active ATM and ATR can phosphorylate the transcription factor SOG1, and thus transcriptionally activate hundreds of genes involved in cell cycle arrest, DNA repair and PCD [14].

The ATP binding cassette (ABC) transporters are engaged in transport of various compounds, including sugars, ions, peptides, and more complex organic molecules [15, 16]. Its 130 members are classified into 8 subfamilies (A-H) in Arabidopsis [17, 18]. While all subfamilies except E and F have nucleotide domain (NBD) and transmembrane domain (TMD), essential for chemical transportation, the functions of subfamilies E and F containing NBD but no TMD remain further elusive [19]. General Control Non-Repressible 20 (GCN20), a member of subfamily F, is soluble ABC protein without TMD [17]. In yeast, GCN20 promotes the kinase activity of GCN2, which is required for yeast growth under amino acid starvation [20]. In Arabidopsis, the mutation in *GCN20* impairs pathogen associated molecular patterns-triggered stomatal closure [21].

In this study, we report that GCN20 is involved in root growth as a novel factor for DNA damage repair. Our results indicated that *gcn20–1* plant has short primary roots and the mutant phenotype can be rescued by expressing *GCN20* in *gcn20–1*. Further experiments demonstrated that the mutation in GCN20 impairs the DNA damage repair since the mutant is hypersensitive to MMC, MMS and UVC. Thus, GCN20 is involved in root elongation by modulating DNA damage repair.

## Results

### *GCN20* positively regulates root meristem growth

**Fig. 1:**
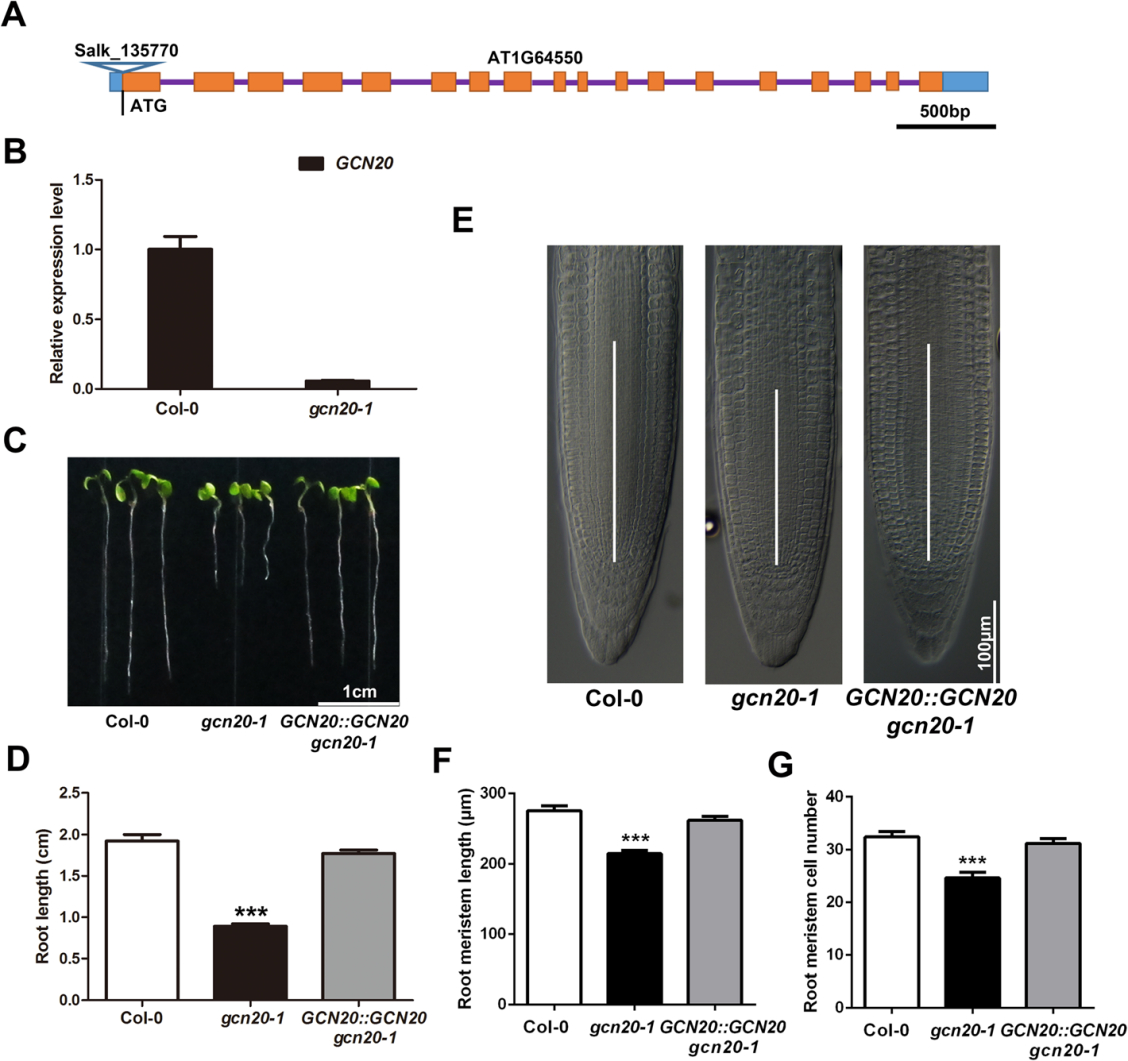
The *gcn20–1* mutant displays reduced root growth. **a** The diagram shows T-DNA insertion site in *GCN20* (At1g64550) of Salk_135770 (*gcn20–1*). Rectangles represent exons and lines between the exons stand for introns. **b** The expression level of *GCN20* was assayed by qPCR in the wild type and *gcn20–1*. The expression level of the wild type is set to 1. *ACT2* was used as an internal control. **c** The representative images of the 6-d-old wild-type, *gcn20–1* and *GCN20::GCN20 gcn20–1* seedlings. Bar = 1 cm. **d** Roots length of 6-day-old wild-type, *gcn20–1* and *GCN20::GCN20 gcn20–1* seedlings. **e** The photographs for the root meristem regions of 6-day-old wild-type, *gcn20–1* and *GCN20::GCN20 gcn20–1* plants. Bar = 100 μm (**f, g**) Size (**f**) and cell number (G) of the root meristem of 6-day-old wild-type, *gcn20–1* and *GCN20::GCN20 gcn20–1* seedlings. Data shown are means ± SEM. Asterisks indicate significant differences with respect to each control (Student’s *t* test): ***, *P* < 0.001

To investigate possible role of *GCN20* in plant growth, we identified a T-DNA insertion allele (Salk_135770) from the Arabidopsis Biological Resource Centre as *gcn20–1*. PCR-based genotyping by flanking the insertion indicated that T-DNA inserts at − 43 bp upstream of the translation start site of *GCN20* (Fig. 1a). Quantitative PCR (qPCR) analysis showed that the expression of *GCN20* in the mutant was severely repressed (Fig. 1b). Then, we examined the growth of the mutant by measuring root lengths and found that the primary root of the mutant was shorter than that of the wild type (Fig. 1c, d), indicating a positive role of *GCN20* in root growth. To further confirm it, we introduced the genomic sequences of *GCN20* including its promoter into *gcn20–1* plants and assayed primary root lengths, As expected, *GCN20::GCN20 gcn20–1* lines have similar root lengths as the wild type did. (Fig. 1c, d).

**Fig. 2:**
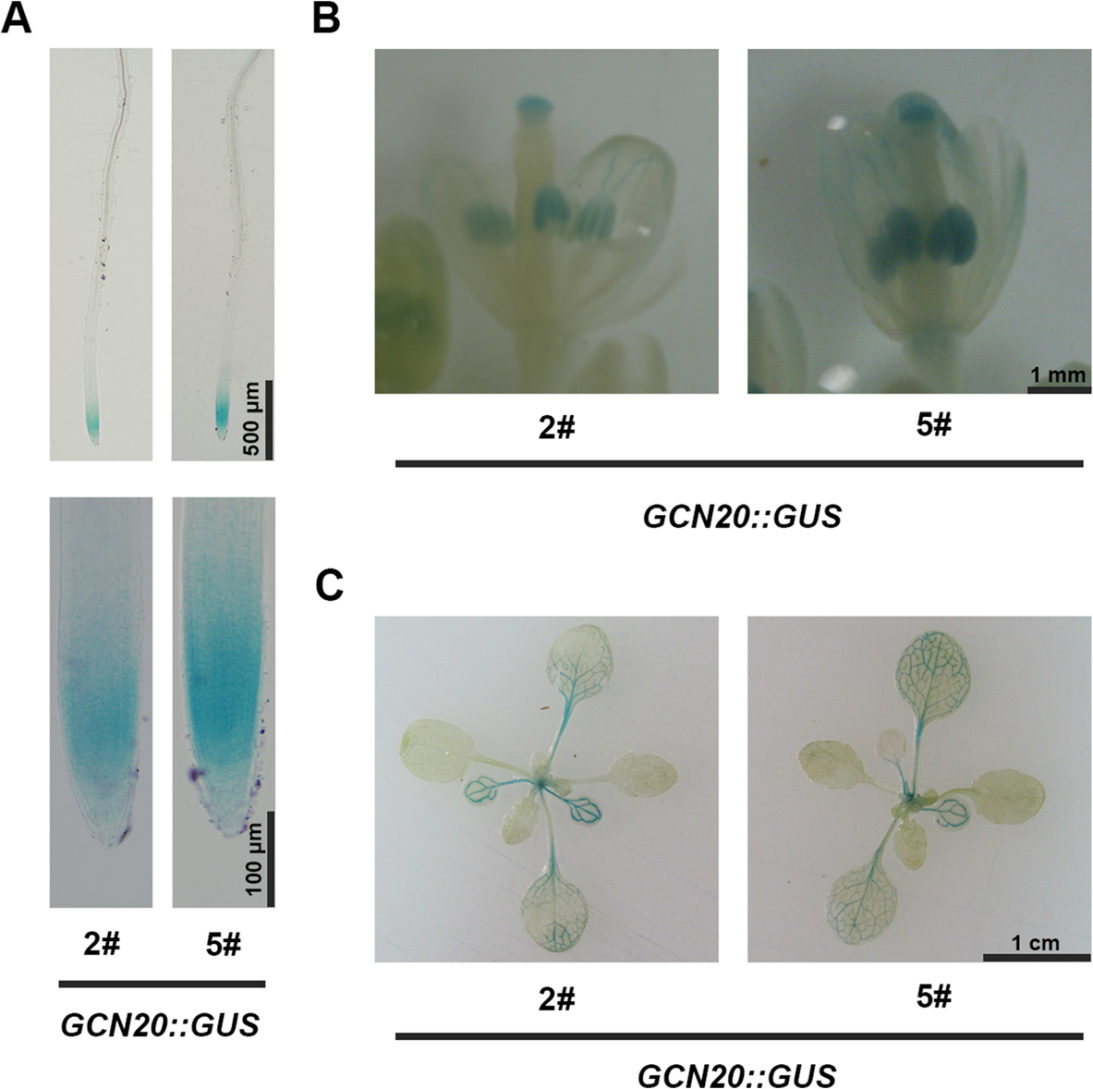
The expression pattern of *GCN20*. **a**-**c** GUS staining of *GCN20::GUS* plants in roots (**a**), anthers and stigmas (**b**) and young seedlings (**c**). 2# and 5# stand for two independent lines

Shorter root is usually associated with changes of the meristem region as indicated in previous reports [22, 23]. Thus, we focused on the root meristem zone of the mutant. Indeed, decreased meristem length and cell number were assayed in *gcn20–1* roots compared with those in the wild type and *GCN20::GCN20 gcn20–1* (Fig. 1e-g), suggesting that *GCN20* affects root length by regulating root meristem. To function in root meristem, *GCN20* may express in root. Thus, we obtained transgenic lines *GCN20::GUS* and examined the spatial expression of *GCN20* via GUS-staining. Our data showed that *GCN20* expressed in the root meristem (Fig. 2a). In addition, GUS activity was also detected in other tissues such as anthers, stigmas and leaves. (Fig. 2b, c).

### The *gcn20–1* shows increased cell cycle arrest at the G2/M checkpoint

**Fig. 3:**
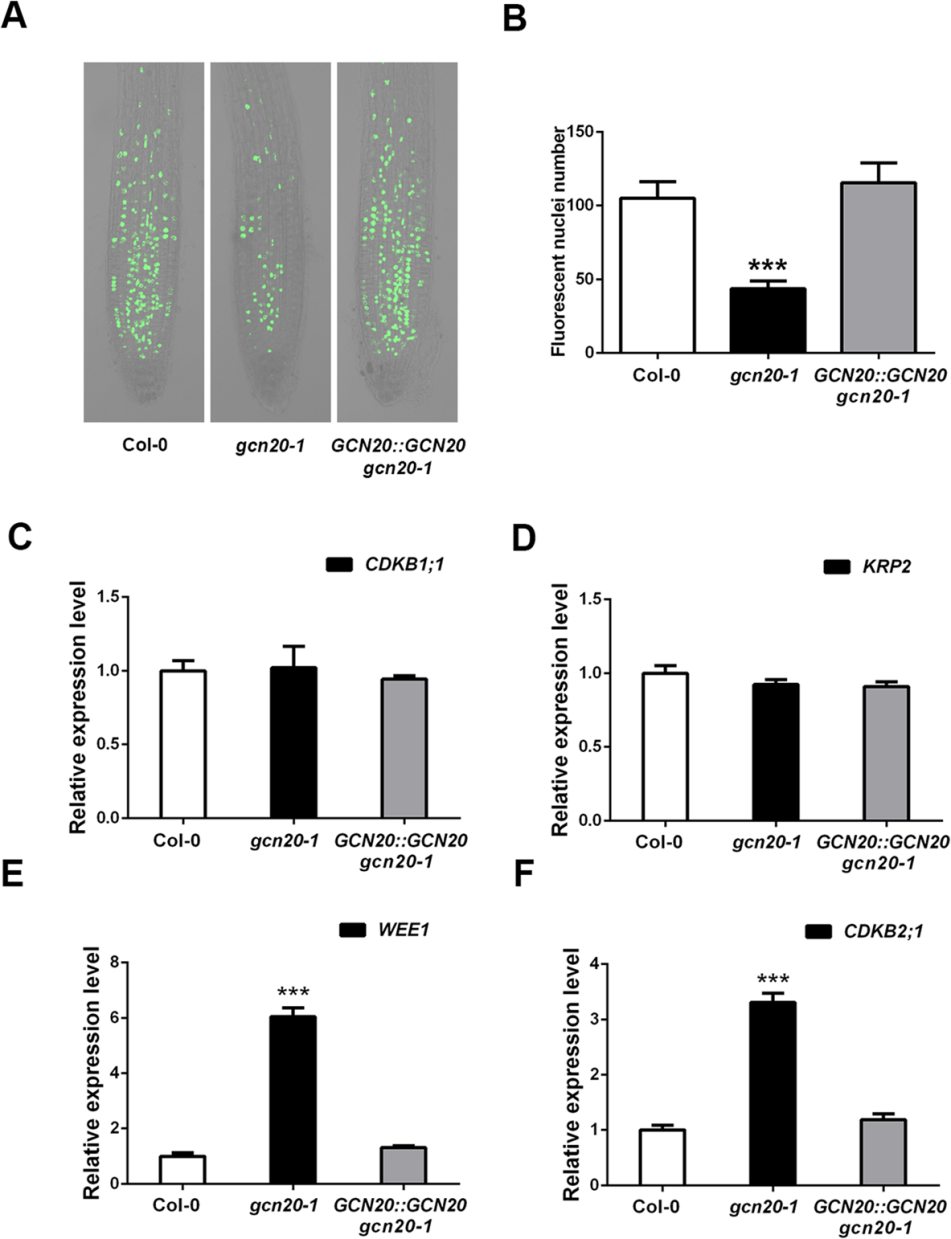
The mutation in *GCN20* results in cell cycle arrest at G2/M phase. **a** Analysis of cellular DNA replication in wild-type, *gcn20–1* and *GCN20::GCN20 gcn20–1* plants by an EdU incorporation assay. **b** Fluorescence nuclei number of wild-type, *gcn20–1* and *GCN20::GCN20 gcn20–1* root meristems in EdU incorporation assay. **c**-**f** The expression of *CDKB1;1* (**c**), *KRP2* (**b**), *WEE1* (**e**) and *CDKB2;1* (**f**) were assayed by qPCR in the wild type, *gcn20–1* and *GCN20::GCN20 gcn20–1*. The expression level of the wild type is set to 1 and *ACT2* was used as an internal control. Data shown are means ± SEM. Asterisks indicate significant differences with respect to each control (Student’s *t* test): ***, *P* < 0.001

It has been reported that reduced root meristem could be due to the deficiency in cell cycle regulation [24]. Thus, we determined cell dividing in the root meristem of *gcn20–1* by utilizing a nucleotide analog 5-ethynyl-2′-deoxyuridine (EdU) incorporation assay, which shows actively replicating cells [25]. We found that the number of actively replicating cells in root meristems was significantly reduced in *gcn20–1* compared with the wild type and *GCN20::GCN20 gcn20–1* (Fig. 3a, b), implying cell cycle arrest occurring in the root meristems of *gcn20–1* plants. Then, we further analyzed the expression of the cell cycle related marker genes: *CDKB1;1* (specifically activated in early S phase and M phase), *KRP2* (a negative regulator of G1/S checkpoint), and *WEE1* and *CDKB2;1*, which trigger G2/M checkpoint [7]. Our results exhibited that the expressions of *KRP2* and *CDKB1;1* were similar in the roots of wild-type, *gcn20* and *GCN20::GCN20 gcn20–1* plants, whereas the expression of *CDKB2;1* and *WEE1* was significantly upregulated in the mutant compared with that in the wild type and *GCN20::GCN20 gcn20–1* (Fig. 3c-f), suggesting that *GCN20* is involved in G2/M checkpoint.

### *GCN20* is involved for DNA damage repair

**Fig. 4:**
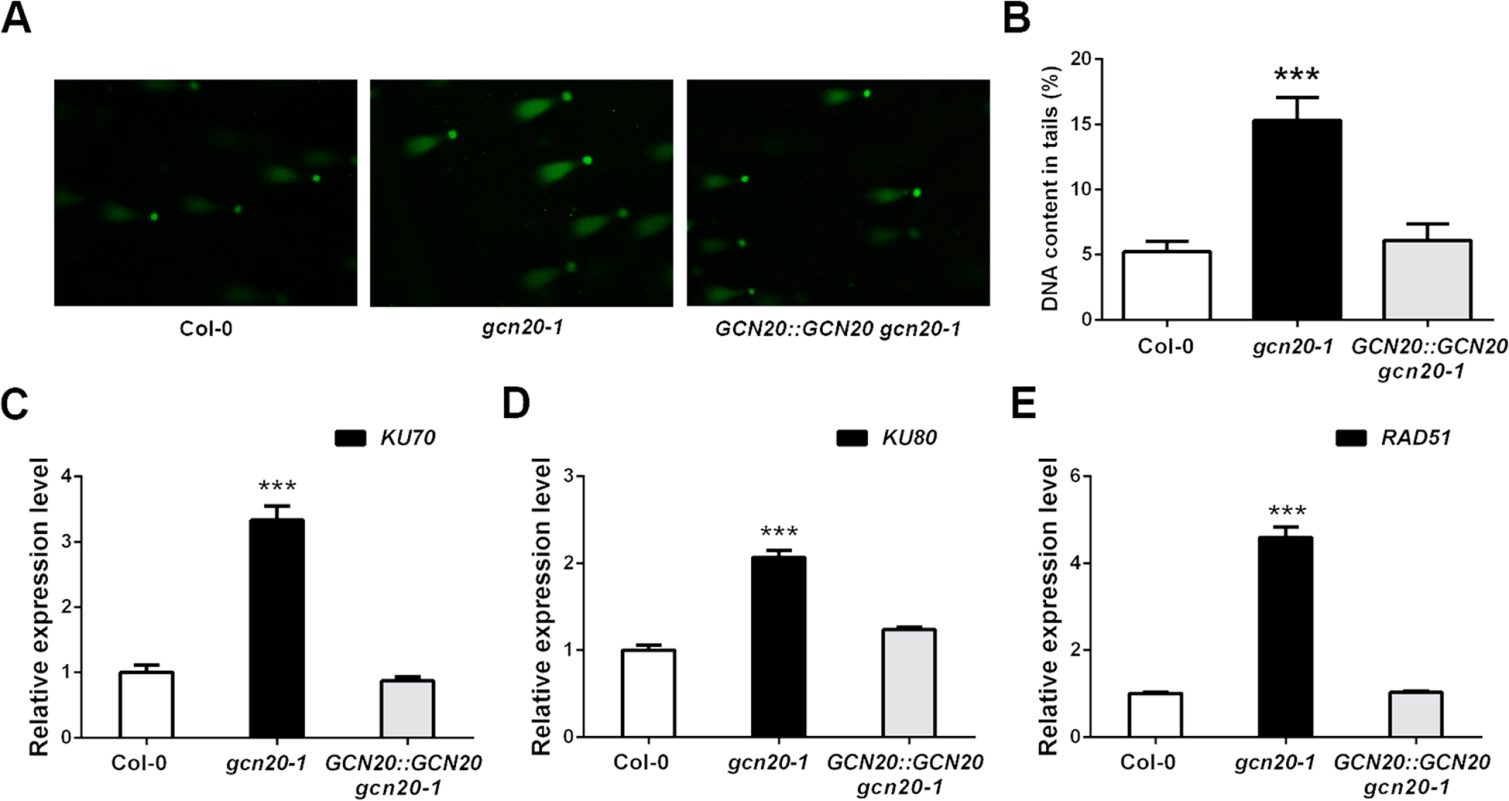
DNA damage analysis of *gcn20–1* mutant. **a** Analysis of DNA damage in wild-type, *gcn20–1* and *GCN20::GCN20 gcn20–1* plants by comet assay. **b** Quantitative analysis of the DNA in tails of wild-type, *gcn20–1* and *GCN20::GCN20 gcn20–1* plants in comet assay. **c**-**e** The expression of *KU70* (**c**), *KU80* (**d**) and *RAD51* (**e**) was assayed by qPCR in the wild type, *gcn20–1* and *GCN20::GCN20 gcn20–1.* The expression level of the wild type is set to 1 and *ACT2* was used as an internal control. Data shown are means ± SEM. Asterisks indicate significant differences with respect to each control (Student’s *t* test): ***, *P* < 0.001

The G2/M checkpoint is also known as G2/M DNA damage checkpoint [7, 26], which ensures cells to repair DNA damage before entering mitosis. Since our data above suggest a role of *GCN20* in G2/M checkpoint, we further evaluated the DNA damage with a comet assay [27]. Indeed, the percentage of DNA in the tail of *gcn20–1* cells was significantly higher than that in the wild type and *GCN20::GCN20 gcn20–1* (Fig. 4a, b), indicating that *gcn20–1* has more damaged DNA. To further confirm this, we analyzed the expression of several DNA damage-induced genes: *KU70*, *KU80* and *RAD51* [28, 29]. Our qPCR analysis showed that all of these three genes were significantly upregulated in *gcn20–1* in comparison with those in the wild type and *GCN20::GCN20 gcn20–1* (Fig. 4c-e), further supporting that the mutation in *GCN20* results in higher accumulation of damaged DNA.

**Fig. 5:**
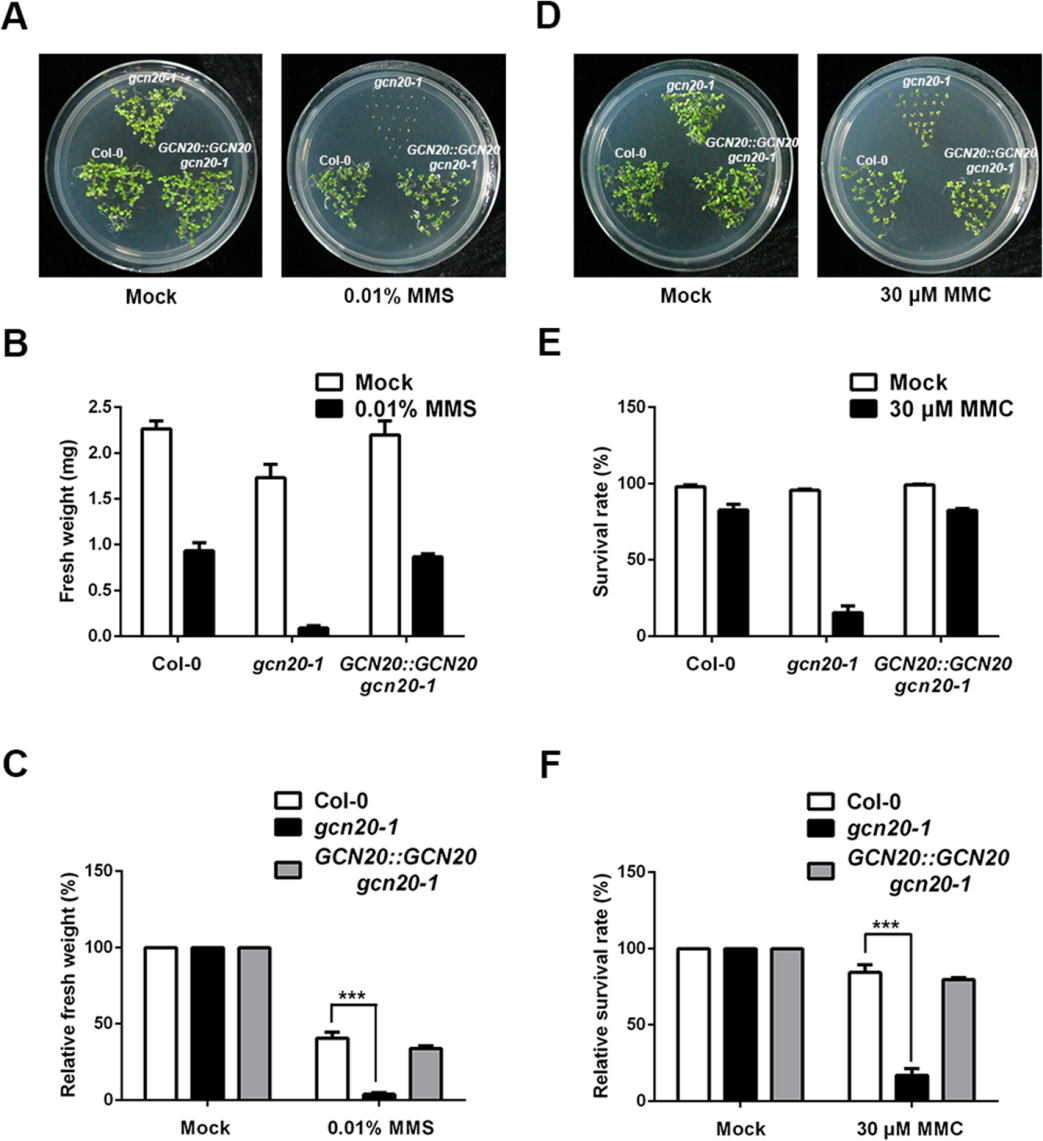
The *gcn20–1* mutant is sensitive to chemically induced DNA damage. **a** The wild type, *gcn20–1* and *GCN20::GCN20 gcn20–1* were grown with or without 0.01% MMS for 12 days before photographed. **b** Quantitative analysis of fresh weights of wild-type and *gcn20–1* and *GCN20::GCN20 gcn20–1* plants grown with or without 0.01% MMS for 12 days. **c** Relative fresh weights of wild-type, *gcn20–1* and *GCN20::GCN20 gcn20–1* plants treated with 0.01% MMS for 12 days compared with their untreated control. **d** The wild type and *gcn20–1* and *GCN20::GCN20 gcn20–1* were grown with or without 30 μM MMC for 12 days before photographed. **e** Quantitative analysis of survival rates of wild-type and *gcn20–1* and *GCN20::GCN20 gcn20–1* plants grown with or without 30 μM MMC treatment for 12 days. **f** Relative survival rates of wild-type, *gcn20–1* and *GCN20::GCN20 gcn20–1* plants treated with 30 μM MMC for 12 days compared with their untreated control. Seedlings were scored survival when plants had two or more true leaves. Data shown are means ± SEM. Asterisks indicate significant differences with respect to each control (Student’s *t* test): ***, *P* < 0.001

The higher accumulation of damaged DNA in *gcn20–1* could be due to impaired ability of DNA repair [11]. Thus, we further tested the sensitivity of *gcn20–1* to DNA damage inducing agent methyl methanesulfonate (MMS) and mitomycin C (MMC), which has been wildly used to verify gene function in DNA damage repair [7, 30–32]. When treated with DSB-inducing agent MMS, the *gcn20–1* mutant was much more sensitive than the wild type and *GCN20::GCN20 gcn20–1* plants in term of changes in fresh weights of treated *gcn20–1*, wild-type and *GCN20::GCN20 gcn20–1* plants compared with untreated controls, respectively (Fig. 5a-c). We also assessed the survival rates of wild-type, *gcn20–1* and *GCN20::GCN20 gcn20–1* plants treated with inter-strand crosslinking agent MMC. The survival rates were up to 84.47% for wild-type plants but only 17.03% for *gcn20–1* plants when subjected to 30 μM MMC (Fig. 5d-f), further revealing that the ability of DNA damage repair is impaired in *gcn20–1*. Taken together, our data our data suggest that *GCN20* is involved in DNA damage repair.

### The *gcn20–1* mutant is sensitive to UV light

**Fig. 6:**
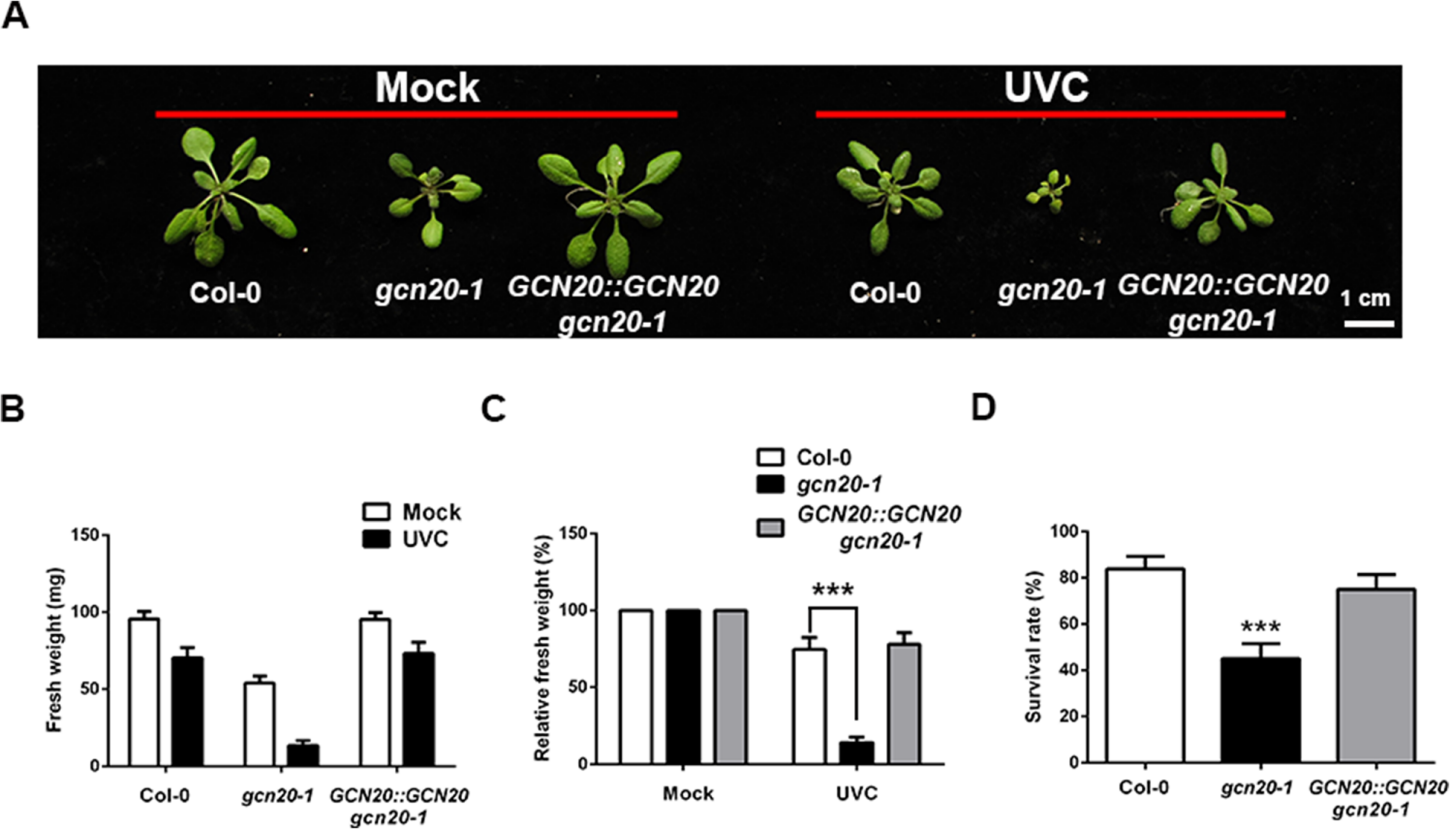
The *gcn20–1* mutant is sensitive to UVC. **a** 3-week-old seedlings of the wild type, *gcn20–1* and *GCN20::GCN20 gcn20–1* grown in white light or white light with UVC. Bar = 1 cm (**b**) Quantitative analysis of fresh weights of wild-type, *gcn20–1* and *GCN20::GCN20 gcn20–1* plants treated with or without UVC shown in (**a**). **c** Relative fresh weights of wild-type, *gcn20–1* and *GCN20::GCN20 gcn20–1* plants treated with UVC compared with their untreated control. **d** Quantitative analysis of survival rates of 2-week-old wild-type, *gcn20–1* and *GCN20::GCN20 gcn20–1* plants treated with high dose (8 KJ/m^2^) of UVC for 3 h. Survival rates were analyzed after 7 days recovery. Data shown are means ± SEM. Asterisk indicates significant differences with respect to its control (Student’s *t* test): ***, *P* < 0.001

It is well-known that UV inhibits plant growth by inducing DNA damage [33]. Therefore, we also examined whether *gcn20–1* with impaired ability of DNA repair is more sensitive to UV treatment. For this purpose, we assayed both fresh weight and survival rates of *gcn20–1* plants treated with UVC. While fresh weight of treated *gcn20–1* was reduced to 14.36% compared with untreated control, up to 74.73% fresh weight of wild-type seedlings was assayed (Fig. 6a-c), indicating that the effect of UVC treatment is more pronounced in *gcn20–1* seedlings compared with wild-type plants. Similarly, the wild type had much higher survival rate (83.75%) than 45.13% of *gcn20–1* seedlings when treated with high dose UVC (Fig. 6d). These data suggested that impaired ability of DNA repair in *gcn20–1* is responsible for higher sensitivity of *gcn20–1* to UVC treatment in comparison with the wild type.

## Discussion

The ATP binding cassette transporters have been implicated in the transport of various compounds [16]. However, the members of its subfamilies E and F do not contain transmembrane domain, which is essential for chemical transportation [17]. Thus, the function of these subfamilies remains enigmatic. Arabidopsis GCN20, a member of subfamily F, shares sequence similarities with yeast (46% identity and 66% similarity) and mammalian (41%identity and 61% similarity) GCN20 proteins (EDN59158 and NP_038880, respectively) [21]. Previous reports showed that yeast GCN20 promotes the kinase activity of GCN2 [20] and the mutation in *GCN20* impairs pathogen associated molecular patterns-triggered stomatal closure [21]. Here, we demonstrated that GCN20 is involved in primary root elongation by regulating meristem size and cell number.

Root growth is determined by meristem cell division and subsequently cell elongation/differentiation [22]. In the root meristem, the meristematic activity of stem cells is modulated by both developmental and environmental factors [24]. Our study showed that the *gcn20–1* plants have shorter primary roots with shorter meristem size and reduced cell number than the wild type, indicating a positive role of GCN20 in root growth. Consistent its function in root growth, *GCN20* expresses in root tip including root meristem. This gene is also expressed in leaves, consistent with the finding by Zeng et al. that *gcn20–1* plants have pale green leaves [21].

Cell cycle arrest in root meristem impairs plant growth and development [8]. Cell cycle arrest can be restrained by checkpoints and cell cycle checkpoints provide the cells with sufficient time to either cope with the damaged DNA or undergo cell death [14, 34]. In particular, the G2/M checkpoint allows cells to repair replication errors and damages before proceeding into mitosis [6]. Our EdU incorporation assay showed that the number of actively replicating cells in *gcn20–1* root meristems is reduced compared with the wild type. Further, the expression of *CDKB2;1* and *WEE1* is significantly upregulated in the mutant compared with that in the wild type, suggesting that GCN20 is involved in G2/M checkpoint.

Since DNA damage leads to a cell cycle arrest, DNA damage repair also modulate the root meristem cell division [26]. For example, RCC1/UVR8/GEF-Like 3 (RUG3) modulates root meristem activity by regulating DDR, and cell cycle progression [7]. We indicated that GCN20 functions in root growth. The NBDs of the ABC proteins, necessary for chemical transport, are engaged in ATP binding and cleavage [17]. This domain is present not only in ABC transporters but also in a variety of non-transporter ABC proteins. Many non-transporter ABC proteins such as Radiation sensitive 50 (Rad50) and Mutator gene S (MutS) are involved in DNA repair [35]. In our study, we also suggest that non-transporter ABC protein GCN20 is involved in DNA repair as *gcn20–1* accumulates more damaged DNA.

It is reported that UVC, MMS and MMC trigger different types of DNA damage [30, 36–38]. UVC brings cyclobutane pyrimidine dimers in DNA, MMS is DSB-inducing agent and MMC triggers inter-strand crosslinking. In Arabidopsis, ATM and ATR function as main DDR regulators to coordinate cell cycle progression and the activation of DNA repair pathways [4]. While DSBs trigger ATM activation, UV photoproducts, DNA breaks and DNA crosslinks promote ATR activation. Both ATM and ATR phosphorylate the transcription factor SOG1, and thus transcriptionally activate the expression of hundreds of genes involved in DNA repair [6, 39]. Our results showed that *gcn20–1* was more sensitive to all the three agents (MMC, MMS and UVC), suggesting that GCN20 may function downstream of ATM/ATR pathways.

## Conclusion

In summary, GCN20 functions in root growth by changes of damaged DNA accumulation probably through DNA damage repair. Our study will be useful to understand the functions of non-transporter ABC proteins in plant growth. However, further studies are required to reveal the molecular mechanism of GCN20 in DNA damage repair.

## Methods

### Plant materials and growth conditions

The line Salk_135770 was obtained from Arabidopsis Biological Resource Centre. *Arabidopsis thaliana* seeds were surface sterilized with 5% chloros for 5 min, washed three times with sterile water, placed at 4 °C for 3 days, and then planted on 1/2 MS medium [40] with 0.8% agar and 1% sucrose, pH 5.8 at 23 °C and 100 μmol m^− 2^ s^− 1^ illumination under 16 h light/8 h dark conditions [41, 42].

### Vector constructs and transgenic lines

To make *GCN20::GCN20* construct, the 8 kb genomic sequence of Arabidopsis *GCN20* including 3 kb upstream sequence of start codon and 1 kb downstream sequence of stop codon was amplified using PCR with specific primers, inserted into pCambia1300 vector at *Bam*H I site and confirmed by sequencing. For *GCN20::GUS* construct, the 3 kb upstream sequence of *GCN20* start codon was inserted into *Bam*H I/*Nco* I-digested pCambia1301. *GCN20::GCN20* construct was transformed into *gcn20–1* mutant and *GCN20::GUS* construct into the wild type by *Agrobacterium tumefaciens* strain GV3101 using the floral dip method as we previously reported [22]. Primers used in this study were listed in Additional file 1: Table S1.

### Measurement of root length

Seeds were germinated on 1/2 MS medium as described above and grown in a vertical position. Digital images of seedlings were captured for subsequent measurement of the lengths of roots, Root lengths were measured by using a line traced along the root.

### Measurement of root meristem size and cell number

Measurement were performed according to our previously described method [43]. Seeds were germinated on half-strength MS medium containing 1% sucrose and 0.8% agar and grown in a vertical position. The number of root meristem cells was defined by counting the number of cells in a file extending from the initial cell adjacent to the QC to the first elongated cell in the cortex layer. Meristem size was measured from the QC to the first elongated cell in the cortex layer. Results presented are averages of more than 30 seedlings and experiments were repeated at least three times.

### Microscopic analyses

For the observation of the root meristem zone and GUS staining, the seedlings were mounted with clearing solution (8 g of chloral hydrate, 2 ml of water and 1 ml of glycerol) on glass slides, examined under an Olympus BX60 differential interference contrast (DIC) microscope and photographed by a charge-coupled device (CCD) Olympus dp72 [44].

Confocal microscopy was performed using an Olympus FluoView 1000 confocal laser-scanning microscope according to the manufacturer’s instructions. 20 μg ml^− 1^ propidium iodide (PI) staining were used in PI staining assay.

### RNA extraction and gene expression analysis

RNA extraction and qPCR were performed according to our previously described method [43]. Total RNA extraction was performed using PureLink™ Plant RNA Reagent (Invitrogen) according to the manufacturer’s instruction. RNA samples were then treated with RQ1 RNase-free DNase I (Promega) to remove DNA. The reverse transcription was carried out by using ReverTra Ace® (Toyobo). RNA was quantified by Qubit 3.0 Fluorometer nucleic acid detector (Life). 200 ng total RNA added in each reverse transcription reaction. qPCR assay was performed by using a CFX96™ Real-Time PCR Detection System (Bio-Rad) with ACT2 (AT3G18780) as the reference gene. No-template controls were performed for each pair of primers. PCR was performed as follows: 3 min at 95 °C, followed by 40 cycles of denaturation for 15 s at 95 °C, annealing for 15 s at 58 °C and extension for 20 s at 72 °C. Relative expression was analyzed by ΔΔC(t). Melt curves analysis were assigned as follows: 65 °C to 95 °C, increment 0.5 °C. Efficiency of reactions were considered to 100%. All experiments were performed with three independent biological replicates and three technical repetitions. The genes specific primers used are listed in Additional file 1: Table S1.

### GUS staining

GUS staining was performed based on the procedures as previously described [23]. In brief, plants were incubated in staining solution: 100 mM sodium phosphate buffer, pH 7.5, containing 1 mM 5-bromo-chloro-3indolyl-β-D-glucuronide, 0.5 mM K3[Fe(CN)6] and 0.5 mM K4[Fe(CN)6], 10 mM EDTA, pH 8.0 and 0.1% Triton X-100 at 37 °C. The staining time depended on different tissues of the transgenic lines.

### EdU incorporation assay

EdU incorporation assay was performed as previously described [25]. The wild-type and gcn20–1 plants were grown on 1/2 MS agar plate for 5 days and then incubated in 1/2 MS liquid medium with 10 μM 5-Ethynyl-2′-deoxyuridine (EdU) (Invitrogen, Carlsbad, CA, USA) for 30 min. Then the seedlings were fixed for 30 min in 4% (*w*/*v*) formaldehyde solution in phosphate buffered saline (PBS) with 0.1% Triton X-100. Following 3 × 10 min PBS washes, the seedlings were incubated for 30 min at room temperature in EdU detection cocktail (RiboBio, Cell-Light™ Apollo stain Kit) followed by a 10 min rinse. Finally, the root tips of wild-type and *gcn20–1* seedlings were imaged with Laser-scanning confocal microscope using the Argon laser 488 nm excitation and 478–553 nm emission lines. The fluorescent nuclei represent actively incorporating (replicating) nuclei.

### Comet assay

Comet assay was performed as described by Menke et al. [36]. About 75–150 mg of the plant material were prepared for the comet assay. The comet assay was performed in a darkroom with dim red light. Microscopic slides were precoated with a layer of 1% normal melting point agarose and thoroughly dried at 60 °C. The seedlings were sliced with a razor blade in 300–400 μl PBS (160 mM NaCl, 4 mM NaH_2_PO_4_, 8 mM Na_2_HPO_4_, pH 7.0) containing 50 mM EDTA. Two drops of 30 μl nuclei suspension were dropped separately on each slide, mixed with the same volume of liquid 1% low melting point agarose at 42 °C and covered with a coverglass. Nuclei were then subjected to high salt lysis buffer (2.5 M NaCl, 100 mM EDTA, 10 mM Tris-HCl, pH 7.5) for 20 min at room temperature. Equilibration for 3 × 5 min in 1 × TBE (90 mM Tris-borate, 2 mM EDTA, pH 8.4) buffer on ice was followed by electrophoresis at room temperature in 1 × TBE buffer at 30 V (1 V/cm, 15–17 mA) for 6 min. The comets were visualized by staining with SYBR Green I, and then photographed using microscope. At least 50 nuclei for each material were photographed, then the data were analyzed by Casp_1.2.3b2 software.

### Mutagen sensitivity assay

MMS and MMC sensitivity assay were performed according to the previous described [37, 45, 46]. The seeds were plated on 1/2 MS medium with 0.8% agar and 1% sucrose, stratified in the dark at 4 °C for 3 days, and transferred to 1/2 MS medium with 0.8% agar and 1% sucrose containing 0.01% MMS or 30 μM MMC. MMC solutions were made in DMSO at 50 mM for stock. MMS and MMC were added into the medium at approximate 60 °C. The plates were placed in the growth chambers with 16 h light/8 h dark conditions at 23 °C for 12 days and the seedling were used for the assays of fresh weight or survival rate. For fresh weight analysis, the seedlings were wiped off medium and weighed. For survival rate analysis, the seedlings were counted when the plants had two or more true leaves.

### UVC sensitivity assay

UVC sensitivity assay was performed as previously reported by Rosa et al. [30]. UVC were derived from UVC light tube (G13, CnLight), and the dose of UVC were detected by UVC illuminometer (ST-512, SENTRY). For fresh weight analysis, the seedlings were grown in vermiculite under 16 h light/8 h dark at 23 °C for a week. Then, the seedlings were transferred into a chamber under the same growth condition but with additional 2 kJ/m^2^ UVC for additional 2 weeks and used for fresh weight assay. For survival rate analysis, the seedlings were grown in vermiculite under 16 h light/8 h dark at 23 °C for 2 week and then subjected to additional 8 kJ/m^2^ UVC for 3 h. The treated seedling were grown under 16 h light/8 h dark at 23 °C for 1 week recovery and used for survival rates by counting green seedlings as previous report.

### Statistical analysis

All experiments were performed with at least three repetitions. The significance of differences was determined by Student’s *t* test, as indicated in the figure legends.

## Additional file

Additional file 1: **Table S1.** List of the primers used in this study. (DOCX 15 kb)

## Abbreviations

ABC: ATP binding cassette
ATM: Ataxia Telangiectasia mutated
ATR: ATM and Rad3-related
DDR: DNA damage response
DSBs: double strand breaks
EdU: 5-ethynyl-2′-deoxyuridine
GCN20: General Control Non-Repressible 20
MMC: Mitomycin C
MMS: Methyl methanesulfonate
NBD: Nucleotide domain
NBD: Nucleotide domain
PCD: Programmed cell death
SSBs: Single strand breaks
UVC: Ultraviolet C
