# Supplementary material for: General control non-repressible 20 (GCN20) functions in root growth by modulating DNA damage repair in Arabidopsis
Source: BMC Plant Biol. 2018 Nov 12;18:274. doi: 10.1186/s12870-018-1444-9 (PMC6233562; doi:10.1186/s12870-018-1444-9)
Supplement: Supplementary file 1 — Table S1. List of the primers used in this study. (DOCX 15 kb) [file 12870_2018_1444_MOESM1_ESM.docx]

| **Primer Sequence (5’ to 3’)** |
| --- |

**Supporting Table S1.** List of the primers used in this study.

**For qPCR Analysis**

qACT2F CTTGCACCAAGCAGCATGAA

qACT2R CCGATCCAGACACTGTACTTCCTT

qGCN20F ggatggactagacctttcatcg

qGCN20R cagcttttgttctggtactcctg

qKRP2F AGGAGAAGAGAACGAGATGTG

qKRP2R CAGCCACCGAAGAAGAAT

qCDKB1;1F GACCTAATCCTAAGCCTCTTGA

qCDKB1;1R TGCTGCTCAGTTGGTGTTC

qCDKB2;1F CCGGGAAAATCGTCGCTCTA

qCDKB2;1R GGAGAGTGGTGGAAGGAACG

qWEEF TGGTGCTGGACATTTCAGTCGG

qWEER CAAGAGCTTGCACTTCCATCATAG

qRAD51F gagtttggtgtggctgttgttat

qRAD51R aaacatggcgagcttatcactt

qKU70F tatggcgatgaccctgatgaa

qKU70R cgaggagattgttggcagtcag

qKU80F aggagccaaagcaattcaatga

qKU80R ccccagcgttctcgtctactat

**For the *GCN20::GCN20* construct**

GCN20cF agctcggtacccggggatccgctgattgatcagcttgatcatcc

GCN20cR aggtcgactctagaggatccCCATCTTCAGAAAAGCAAAGgtttggtc

**For the *GCN20::GUS* construct**

GCN20pF agctcggtacccggggatccgctgattgatcagcttgatcatcc

GCN20pR CCTCAGATCTACCATagccaacacagaagcgacgagattc

**For the Salk_135770 genotyping**

135770LP GGCGATCATCCTTTTTCTTTC

135770RP TTTTGGGAAAACTGCAATGTC

LBa TGGTTCACGTAGTGGGCCATCG

|  |
| --- |
